# Cytoplasmic expression of laminin-5γ2 chain is associated with progression and acquisition of malignant traits in ovarian serous and mucinous tumors

**DOI:** 10.1097/MD.0000000000049220

**Published:** 2026-06-12

**Authors:** Yosuke Tajika, Johji Imura, Akira Noguchi, Megumi Orita, Yuki Nakajima, Takashi Minamisaka, Kohji Takagi, Tomoko Shima, Akitoshi Nakashima, Kenichi Hirabayashi

**Affiliations:** a Laboratory of Anatomic Pathology, Toyama University Hospital, Toyama, Japan; b Department of Diagnostic Pathology, University of Toyama, Toyama, Japan; c Department of Diagnostic Pathology, Saiseikai Toyama Hospital, Toyama, Japan; d Department of Obstetrics and Gynecology, University of Toyama, Toyama, Japan.

**Keywords:** basement membrane, cytoplasmic expression, laminin-5γ2, malignant transformation, mucinous tumor, ovarian cancer, serous tumor

## Abstract

Ovarian tumors are classified into adenoma, borderline tumor, and adenocarcinoma based on cellular atypia and invasion. However, invasion can be difficult to assess histologically, underscoring the need for objective biomarkers. Laminin-5γ2 chain (Lam5γ2), a component of the basement membrane, has been implicated in tumor cell invasion in various malignancies. This study aimed to investigate the immunohistochemical expression of Lam5γ2 as a potential adjunctive biomarker associated with invasive phenotypes in ovarian serous and mucinous tumors. Lam5γ2 immunohistochemistry was performed on 80 ovarian tumor specimens, including adenomas, borderline tumors, and adenocarcinomas of serous and mucinous subtypes. Lam5γ2 expression patterns were compared across histological categories. In serous tumors, Lam5γ2 expression along the basement membrane was preserved in approximately half of adenomas and borderline tumors but was absent in all adenocarcinomas. Notably, cytoplasmic expression was observed in 11 of 12 serous adenocarcinomas (92%), and cytoplasmic staining was more frequent in borderline tumors and adenocarcinomas than in adenomas. A similar pattern was observed in mucinous tumors, with a decrease in basement membrane expression from adenomas to borderline tumors to adenocarcinomas. In mucinous adenocarcinomas, cytoplasmic expression was present in 6 of 10 cases, while basement membrane linear expression was retained in 3 of 10 cases. These differences were statistically significant in both serous and mucinous tumors (*P* <.001 and *P* = .03, respectively). Loss of Lam5γ2 expression from the basement membrane and aberrant cytoplasmic localization in ovarian serous and mucinous tumor cells were associated with histological progression and invasive phenotypes. Thus, Lam5γ2 immunoreactivity may serve as a potential adjunctive biomarker, particularly in diagnostically challenging borderline or microinvasive cases, pending validation in larger, independent cohorts.

## 1. Introduction

The prognosis for patients with ovarian adenomas is typically favorable; however, ovarian adenocarcinomas are among the most lethal malignancies. According to the World Health Organization classification, there are 14 histological subtypes of ovarian tumors, with epithelial tumors accounting for approximately 90%.^[1]^ Epithelial tumors are further classified into 5 categories: serous, mucinous, endometrioid, clear cell, and Brenner tumors.^[2]^ While serous and mucinous tumors were traditionally believed to originate from the superficial epithelium of the ovary, recent evidence suggests that some serous adenocarcinomas may arise from the tubal epithelium.^[3]^ Each of these ovarian tumors is histopathologically divided into 3 groups – adenoma, borderline tumor, and adenocarcinoma – which closely correlate with clinical patient prognosis.^[2]^ Therefore, histopathological classification plays a crucial role in both prognosis estimation and treatment planning. These 3 tumor categories are currently distinguished based on the degree of cellular atypia and the presence or absence of stromal invasion. A definitive diagnosis of adenocarcinoma requires the identification of stromal invasion. Borderline tumors are characterized by cellular atypia and proliferation in the absence of stromal invasion. However, assessment of invasion is often challenging, and reproducibility may be limited.^[4,5]^

Laminin-5 (Lam5) is a key component of the extracellular matrix and is primarily located along the basement membrane in normal tissues.^[6]^ Its primary function is to anchor epithelial cells to the basement membrane; however, it also plays an important role in cell migration, particularly during wound healing and ulceration.^[7,8]^ In addition, Lam5 regulates several cellular functions through interactions with cell surface receptors.^[9]^ It is composed of 3 short-chain subunits – α, β, and γ – specifically the α3, β3, and γ2 chains.^[9–14]^ Among these, the γ2 chain of Lam5 (Lam5γ2) has been reported to exhibit aberrant expression during the development and progression of various malignant tumors.^[11,15–20]^ Notably, Lam5γ2 is highly expressed in areas of advanced tumor cell invasion,^[16,18,19]^ suggesting its critical role in promoting tumor invasiveness across various organs. In this study, we immunohistochemically investigated the expression of Lam5γ2 to evaluate its potential as a biomarker for the invasive capacity of epithelial ovarian tumors.

## 2. Methods

### 2.1. Case selection

Stored case samples were obtained from the archives of the Diagnostic Pathological Division, University of Toyama Hospital, between January 2005 and December 2014. These cases were preoperatively suspected to be ovarian tumors, for which tumor resection was performed, followed by a pathological diagnosis as serous or mucinous tumors. Specimens were preserved as 10% buffered formalin-fixed, paraffin-embedded sections. The study included 80 Japanese women, aged 18 to 80 years at diagnosis (mean age: 47 years). Specimens were routinely stained with hematoxylin and eosin, then histopathologically classified as serous or mucinous tumors and further categorized as adenomas, borderline tumors, or adenocarcinomas. Among the serous tumors, there were 15 adenomas, 14 borderline tumors, and 12 adenocarcinomas. However, among the mucinous tumors, there were 13 adenomas, 16 borderline tumors, and 10 adenocarcinomas. All pathological slides were independently reviewed by 2 experienced senior pathologists (JI and AN), who were blinded to the clinical data to confirm the diagnoses.

### 2.2. Immunohistochemistry

Immunoperoxidase staining was performed using the GX automated IHC/ISH slide staining system (Roche) according to the manufacturer’s instructions. For immunohistochemical analysis, a mouse monoclonal anti-Lam5γ2 antibody (clone D4B5, MILLIPORE) diluted 1:500 was used as the primary antibody. Antigen retrieval was achieved by enzymatic pretreatment with PROTEASE1 for 8 minutes. Sections were incubated with the primary antibody at 37°C for 48 minutes, followed by incubation with a secondary antibody mixture against mouse and rabbit immunoglobulins at room temperature for 30 minutes (Roche). Immunoreactivity was visualized using the iVIEW DAB Universal Kit (Roche), and the sections were counterstained with hematoxylin. All stained sections were independently reviewed by 2 investigators (YT and JI), who assessed immunoreactivity. In cases of initial disagreement, slides were reevaluated jointly using a double-headed microscope until consensus was reached. Immunoreactivity was assessed based on staining patterns in the basement membrane and/or the cytoplasm of tumor cells. Staining was considered negative when fewer than 5% of tumor cells showed immunoreactivity. This conservative cutoff was selected to avoid overclassification of scattered, equivocal, or nonspecific staining as true tumor cell positivity and to ensure reproducible categorization in small histological subgroups. Interobserver agreement was excellent, with a Cohen kappa coefficient (κ) >0.98.

### 2.3. Statistical analysis

Statistical analyses were conducted using StatFlex version 6 (Artec Co., Japan). Differences in Lam5γ2 expression patterns among adenomas, borderline tumors, and adenocarcinomas in both serous and mucinous tumor types were assessed using contingency table analysis. Because some groups comprised small numbers of cases, Fisher exact test was applied. A *P* value of <.05 was considered statistically significant.

## 3. Results

### 3.1. Serous tumors

**Figure 1.**
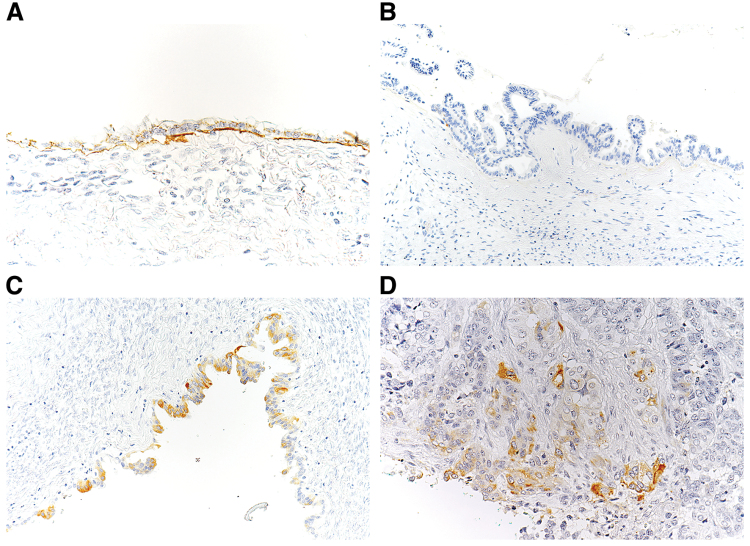
Immunohistochemical findings of laminin-5γ2 chain in serous ovarian tumors. (A) Adenoma showing linear positive staining along the basement membrane. (B and C) Borderline tumor: (B) loss of basement membrane expression; (C) cytoplasmic expression in tumor cells without basement membrane expression. (D) Adenocarcinoma demonstrating cytoplasmic expression in invading tumor cells with reduced or absent basement membrane expression. Original magnification, (A and D) ×20; (B and C) ×10.

In serous tumors, linear Lam5γ2 expression along the basement membrane was observed in 7 of 15 adenomas (47%) (Fig. 1A). Of these, 6 cases (40%) showed preserved basement membrane expression without cytoplasmic staining, while 1 case (7%) demonstrated both basement membrane and cytoplasmic expression. Among the remaining 8 adenomas lacking basement membrane expression, 7 (47%) showed no cytoplasmic staining, and 1 case (7%) exhibited cytoplasmic expression.

Among the borderline tumors, linear basement membrane Lam5γ2 expression was observed in 7 of 14 cases (50%), while the remaining 7 cases (50%) showed complete loss of this expression. Cytoplasmic staining in tumor cells was present in 10 cases (71%). Of the 7 cases with preserved basement membrane expression, 6 (43%) also demonstrated cytoplasmic staining, whereas 1 case (7%) showed no cytoplasmic expression. Among the 7 cases lacking basement membrane expression, 4 (29%) exhibited cytoplasmic staining (Fig. 1C), and 3 (21%) showed neither basement membrane nor cytoplasmic expression (Fig. 1B).

In all serous adenocarcinoma cases, linear basement membrane Lam5γ2 expression was absent. Cytoplasmic staining was detected in 11 of 12 cases (92%), while 1 case (8%) showed neither basement membrane nor cytoplasmic expression. Cytoplasmic localization was particularly prominent in invading tumor cells. Notably, solitary invading cells at the invasive front – often characterized by loss of intercellular adhesion – exhibited strong cytoplasmic expression (Fig. 1D).

The immunohistochemical findings are summarized in Table 1. A statistically significant difference in expression patterns among adenomas, borderline tumors, and adenocarcinomas was observed in serous tumors (*P <*.001).

**Table 1:** Immunohistochemical results for Laminin-5γ2 chain in ovarian serous and mucinous tumors.

Tumor type	Case	Basement membrane linear expression				Overall *P*-value*
		With		Without		
		Cytoplasmic expression		Cytoplasmic expression		
		No	Yes	No	Yes	
Serous tumor, n (%)	41	–	–	–	–	*P* <.001*
Adenoma	15	6 (40)	1 (7)	7 (47)	1 (7)	–
Borderline tumor	14	1 (7)	6 (43)	3 (21)	4 (29)	–
Adenocarcinoma	12	0	0	1 (8)	11 (92)	–
Mucinous tumor, n (%)	39	–	–	–	–	*P* = .03*
Adenoma	13	7 (54)	3 (23)	3 (23)	0 (0)	–
Borderline tumor	16	7 (44)	2 (13)	5 (31)	2 (13)	–
Adenocarcinoma	10	3 (30)	0 (0)	1 (10)	6 (60)	–

* Fisher exact test was used to compare the distribution of the 4 Lam5γ2 expression patterns among adenomas, borderline tumors, and adenocarcinomas within each tumor type. The *P*-values indicate overall group comparisons and are not row-specific. A *P*-value of < .05 was considered statistically significant.

### 3.2. Mucinous tumors

**Figure 2.**
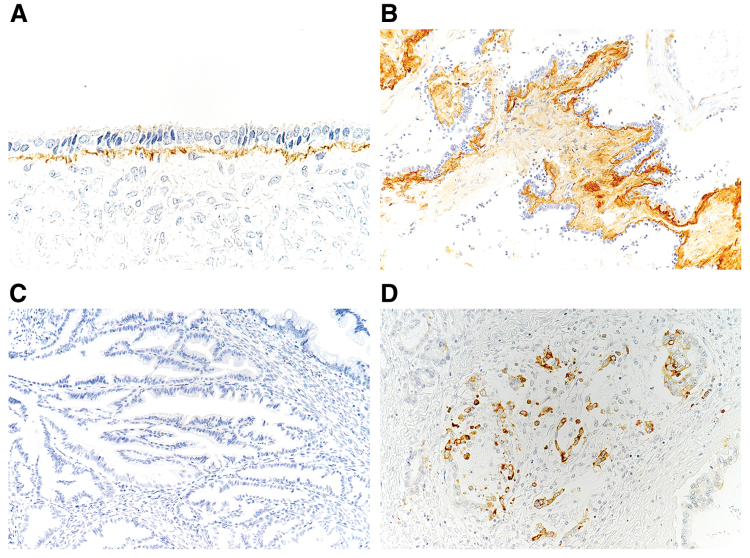
Immunohistochemical findings of laminin-5γ2 chain in mucinous ovarian tumors. (A) Adenoma showing linear positive staining along the basement membrane. (B and C) Borderline tumor: (B) preserved basement membrane expression; (C) complete loss of basement membrane expression. (D) Adenocarcinoma demonstrating cytoplasmic expression in invading tumor cells with reduced or absent basement membrane expression. Original magnification, (A and D) ×20; (B and C) ×10.

In mucinous tumors, 10 of 13 adenomas (77%) exhibited preserved Lam5γ2 expression along the basement membrane (Fig. 2A). Three cases (23%) showed cytoplasmic expression alongside preserved basement membrane staining. Additionally, 3 cases showed loss of basement membrane expression without cytoplasmic expression. Among borderline tumors, 9 of 16 cases (56%) retained basement membrane expression; in 7 of these (44%), cytoplasmic expression was absent (Fig. 2B). Two cases (13%) showed both preserved basement membrane and cytoplasmic expression. In addition, 2 cases (13%) demonstrated cytoplasmic expression accompanied by loss of basement membrane expression. Five cases (31%) showed neither basement membrane nor cytoplasmic expression (Fig. 2C). In mucinous adenocarcinomas, Lam5γ2 was diffusely expressed in the cytoplasm of tumor cells in 6 of 10 cases (60%) (Fig. 2D), whereas basement membrane linear expression was retained in 3 cases (30%). One case (10%) exhibited loss of basement membrane expression without accompanying cytoplasmic expression. The immunohistochemical results are summarized in Table 1. Serous and mucinous tumors showed a progressive pattern of decreasing Lam5γ2 expression along the basement membrane and increasing cytoplasmic expression during progression from adenoma to borderline tumor to adenocarcinoma. A statistically significant difference in expression patterns among adenomas, borderline tumors, and adenocarcinomas was observed in both serous and mucinous tumors (*P* <.001 and *P* = .03, respectively).

## 4. Discussion

Ovarian tumors show a variety of histological subtypes, most of which are epithelial tumors. These epithelial tumors are classified into 5 categories: serous, mucinous, endometrioid, clear cell, and Brenner tumors. Furthermore, they are divided into benign tumors, borderline tumors, and adenocarcinomas to help estimate patient prognosis. Benign and borderline tumors are extremely rare among endometrioid, clear cell, and Brenner tumors, whereas serous and mucinous tumors are most commonly encountered.^[2]^ Correctly distinguishing between benign tumors, borderline tumors, and adenocarcinomas in serous and mucinous types is important not only for patient outcomes but also for selecting appropriate treatment. These 3 types of tumors are diagnosed based on the presence or absence of cellular and histological atypia, invasion, or metastasis.

Notably, borderline tumors are not present in the histological subclassification of tumors in other organs; they have been recognized as being neither adenomas nor adenocarcinomas. This type of tumor is considered an intermediate lesion when only a small number of atypical cells are present or when invasion – an important diagnostic factor – cannot be confirmed. However, objectively evaluating atypia is not always easy, and assessing the presence of invasion and its associated risk is often difficult. Therefore, in this report, we conducted immunohistochemical studies on Lam5γ2 to explore biological markers that may serve as objective methods for evaluating tumor cell invasiveness.

Laminins are a group of molecules comprising more than 16 isoforms that compose the basement membrane. Their functions are diverse, including involvement in cell differentiation, cell adhesion, migration, and structural maintenance of cells.^[10,12]^ Lam5 is composed of 3 short-chain subunits, α, β, and γ, consisting of the α3, β3, and γ2 chains, respectively.^[10–14]^ It was previously recognized as a ligand for the urinary-type plasminogen activator receptor, uPAR, but has recently been identified as a marker of tumor cell invasion in some human malignancies.^[21–25]^ Among these 3 subunits, Lam5γ2 has been reported to show aberrant expression during the development and progression of various malignant tumors.^[11,12,15–20]^ Although Lam5γ2 is normally localized to the basement membrane of epithelial cells in normal tissues, its expression pattern may change in tumor tissue. For example, basement membrane expression may disappear in noninvasive lesions, or Lam5γ2 may appear within the cytoplasm of some tumor cells. Expression tends to be concentrated in sprouting tumor cells within microinvasive lesions. Furthermore, in advanced invasive lesions, this pattern becomes more pronounced, with expression observed in numerous tumor cells at the invasive front.^[18,26]^

In this study, Lam5γ2 expression patterns were compared immunohistochemically among adenomas, borderline tumors, and adenocarcinomas of serous and mucinous tumors. The expression patterns showed similar tendencies across the 3 groups in both tumor types. In adenomas, linear expression was observed along the basement membrane at sites of contact between tumor cells and stroma. In borderline tumors, some cases retained linear basement membrane expression, whereas others demonstrated its loss, with cytoplasmic expression in some tumor cells. In adenocarcinomas, basement membrane expression was frequently absent. Microinvasive lesions showed cytoplasmic expression, consistent with the suggestion that tumor cells were sprouting into the stroma. In advanced invasive lesions, cytoplasmic expression was predominantly observed in tumor cells at the invasive front. Therefore, as malignancy progressed from adenoma to borderline tumor to adenocarcinoma, basement membrane expression became less frequent, whereas aberrant cytoplasmic expression became more frequent. These findings suggest that invasiveness should be regarded as one of the malignant phenotypes of tumor cells in both serous and mucinous tumors. Similar observations have been reported in ovarian mucinous neoplasms: Okuma et al demonstrated that cytoplasmic and stromal expression of the laminin γ2 chain correlated with infiltrative invasion in gastrointestinal-type ovarian mucinous neoplasms.^[27]^ It is possible that this malignant trait is associated with intracellular expression of Lam5γ2. Comparable patterns have been described in other organs. In uterine cervical adenocarcinoma – a gynecological malignancy similar to those examined in the present study – cytoplasmic Lam5γ2 expression was observed as lesions progressed from intraepithelial carcinoma through microinvasive carcinoma to invasive carcinoma.^[24]^ Likewise, a study on Paget disease reported a similar distinction between noninvasive and invasive cases, consistent with the present findings.^[25]^ Furthermore, Lam5γ2 overexpression has also been documented in ovarian clear cell carcinoma, underscoring the broader relevance of altered Lam5γ2 expression in ovarian epithelial tumors.^[28]^ Interestingly, a comparable shift in laminin-5γ2 localization – from the basement membrane to the stroma – has been reported in gallbladder adenocarcinoma. Okada et al demonstrated that stromal expression of laminin-5γ2 was significantly associated with the destructive growth type, characterized by aggressive invasion and poor prognosis.^[29]^ In their study, stromal rather than cytoplasmic or basement membrane staining correlated with lymphovascular invasion, perineural invasion, and a high Ki-67 labeling index. These observations are consistent with the present finding that altered laminin-5γ2 localization in ovarian tumors may be associated with a phenotypic shift related to local aggressiveness and malignant transformation.

The reason Lam5γ2, normally a component of the basement membrane, is expressed in the cytoplasm of tumor cells remains unclear. This displacement may reflect abnormalities in the transcriptional regulation of Lam5γ2 during tumor development, and various signal transduction abnormalities have been reported in this context. β-catenin may induce Lam5γ2 gene expression at the invasive front of tumor cells and in areas showing highly dispersed invasion. Increased Lam5γ2 is secreted extracellularly and cleaved by membrane-type matrix metalloproteinase (MT1-MMP) on the cell surface. This cleavage releases a fragment containing an epidermal growth factor-like sequence. These fragments may regulate autocrine mechanisms that induce tumor cell proliferation and migration by binding to epidermal growth factor receptors on the surface of tumor cells.^[9]^ These observations are consistent with previous reports; however, the present study did not directly examine the underlying molecular mechanisms in ovarian tumors, and this possibility requires further functional validation. In future studies, it will be necessary to investigate whether knockdown of β-catenin using siRNA suppresses Lam5γ2 expression or whether Lam5γ2 expression is enhanced by β-catenin activation.

This study has several limitations. First, it was conducted as a retrospective, single-center analysis using archival specimens, which may restrict the generalizability of the findings. Second, the number of cases in each histological subgroup was relatively small, particularly for certain expression patterns. Third, the study focused exclusively on serous and mucinous tumors, without including other ovarian histological subtypes. Finally, clinical outcome data were not assessed; therefore, the present findings demonstrate associations with histological progression and invasive phenotypes rather than prognostic significance. Future studies with larger, multicenter cohorts and outcome-based validation are warranted.

## 5. Conclusions

As serous and mucinous ovarian epithelial tumors progress from adenoma to borderline tumor to adenocarcinoma, Lam5γ2 expression diminishes along the basement membrane and increasingly appears in the cytoplasm of tumor cells. These findings suggest the potential of Lam5γ2 as an adjunctive biomarker for evaluating malignant progression and identifying invasive phenotypes in serous and mucinous ovarian tumors. Its use should be considered an adjunct to conventional histopathological assessment, particularly in diagnostically challenging borderline or microinvasive cases, pending validation in larger, independent cohorts.

## Acknowledgments

The authors would like to thank Enago (www.enago.jp) for the English language review.

## Author contributions

**Conceptualization:** Yosuke Tajika, Johji Imura.

**Data curation:** Yosuke Tajika, Johji Imura, Akira Noguchi, Takashi Minamisaka, Kohji Takagi, Tomoko Shima, Akitoshi Nakashima.

**Formal analysis:** Yosuke Tajika, Megumi Orita, Yuki Nakajima.

**Investigation:** Yosuke Tajika, Johji Imura, Akira Noguchi, Takashi Minamisaka, Kohji Takagi, Tomoko Shima, Akitoshi Nakashima.

**Methodology:** Yosuke Tajika, Johji Imura.

**Project administration:** Yosuke Tajika.

**Supervision:** Yosuke Tajika.

**Visualization:** Yosuke Tajika, Megumi Orita, Yuki Nakajima.

**Writing – original draft:** Yosuke Tajika, Johji Imura, Akitoshi Nakashima, Kenichi Hirabayashi.

**Writing – review & editing:** Yosuke Tajika, Johji Imura, Akitoshi Nakashima, Kenichi Hirabayashi.
